# From radiation beams to digital streams: social media engagement of German radiotherapy facilities

**DOI:** 10.1007/s00066-025-02439-3

**Published:** 2025-07-17

**Authors:** Fatima Zahra Ouahmane, Ann-Katrin Exeli, Linda Agolli, Daniel Habermehl

**Affiliations:** Department of Radiotherapy, Wilhelm-Conrad-Roentgen-Clinic, University Hospital Giessen-Marburg, Giessen, Germany

**Keywords:** Content strategy, Healthcare communication, Content analysis, Institutional accounts, Online presence

## Abstract

**Purpose:**

Social media (SoMe) increasingly impacts communication in the healthcare sector. Despite its growing relevance, there is limited research on how these institutions utilize SoMe for their purposes. We conducted a descriptive analysis with the aim of evaluating the presence, frequency, and content focus of SoMe activities of German radiotherapy institutions.

**Methods:**

The study analyzed SoMe activities of German radiotherapy institutions. Institutions were identified using selected keywords, and their presence on LinkedIn, X, Instagram, YouTube, and Facebook was recorded. The analysis assessed platform activity, posting frequency, content categories, and follower distribution, distinguishing between hospital and medical care center types, over a 6-month period. Contents were categorized into six themes, including clinical updates, research, events, job postings, education, and team news.

**Results:**

The analysis showed that a total of 24% of German radiotherapy institutions had active social media profiles, with university and larger hospitals being more active than smaller practices or medical care centers (MVZ, *medizinische Versorgungszentren*). The majority of content focused on events and announcements (34%), followed by health awareness and education (19%), research findings (16%), and team news (15%). Job postings (8%) and clinical updates (7%) were less frequently shared. Instagram has the largest follower share (46%), but LinkedIn remains the most active platform, accounting for 86% of posts.

**Conclusion:**

LinkedIn emerges as the leading platform for institutional presence, engagement, and content distribution, with a strong focus on events, scientific studies, and team news; while Instagram leads in follower count, LinkedIn dominates in posting activity.

**Supplementary Information:**

The online version of this article (10.1007/s00066-025-02439-3) contains supplementary material, which is available to authorized users.

## Introduction

Social media (SoMe) is an indispensable tool for communication, both in the private and professional sphere. Platforms such as LinkedIn, X (formerly Twitter), and Facebook play a growing role in the closer communication with patients, the recruitment of specialists, and the dissemination of scientific knowledge [1]. In the professional medical community, the internet in general and SoMe support the rapid exchange of growing medical knowledge in terms of scientific updating, surveys, networking, and case-based learning [2–7]. More and more medical organizations are publishing recommendations for SoMe users in a professional context, especially regarding the promotion and sharing of medical and scientific content [8, 9]. Platforms like LinkedIn, Twitter, and YouTube connect professionals, share updates, promote cancer prevention, and highlight clinical trials.

Radiotherapy facilities, including clinics, medical practices, medical care centers, and university hospitals, face the challenge of strategically shaping their digital design and presence, while at the same time taking readers into a sensitive specialist field [1, 10]. A 2018 study of SoMe activity in radiation oncology societies such as the American Society for Radiotherapy and Oncology (ASTRO) and the European Society for Radiation Oncology (ESTRO) found that most posts focused on social events, with only 10% dedicated to scientific content [11].

Our analysis is intended to contribute to a better understanding of the status of SoMe use in radiotherapy institutions in Germany. The focus of the current report was on the frequency of postings and their content. The posts were grouped thematically into categories such as news, recruitment, information on treatment options, and the dissemination of new publications. In addition, the use of SoMe was compared between types of institutions in order to find differences and trends. To our knowledge, this is the first detailed analysis focusing specifically on SoMe content and platform usage patterns of German radiotherapy institutions.

## Methods

Accounts were identified using a two-step approach. First, we used the list of institutions available on the official website of the German Society of Radiation Oncology (DEGRO, *Deutsche Gesellschaft für Radioonkologie*; degro.org) [9] and conducted a search by entering their official names (i.e., we searched for the name of each institution directly). During this process, we observed that not all official institutional names corresponded exactly to the names used on social media platforms or that the institutions sometimes used English names instead of their German counterparts. To support our search strategy, we supplemented it by entering relevant keywords such as “radiation oncology,” “radiotherapy,” “*Strahlentherapie*,” and “cancer center,” along with the filter for institutions located in Germany. To support data transparency and facilitate reuse, we provide an overview in the supplementary material listing all identified institutional profiles across LinkedIn, Facebook, Instagram, YouTube, and X, including direct hyperlinks to each verified account (Supplementary Table 1).

In order to define what constitutes an “official institutional account,” we established that the name found on the platform, whether it matched the official institutional name or not, had to clearly refer to the physical institution and not to a personal or loosely affiliated account. To ensure this, we examined the account information and verified that it was indeed linked to the official institution. For example, on LinkedIn, we reviewed the account description, affiliations, and other available metadata to confirm the account’s authenticity as an institutional presence.

The geographical and proportional distribution of radiotherapy institutions across German federal states is illustrated in supplementary figures 1 and 2. The number of active institutions was then recorded for the five best known SoMe platforms (LinkedIn, X, Instagram, YouTube, and Facebook). The analysis focused on the previous 6 months, with the most recent data accessed on November 11, 2024, and examined the following key aspects:

1. During the manual data collection process, we simultaneously observed the activity of each account to determine whether it was actively maintained. The presence and activity of radiotherapy institutions were measured using both relative values (percentage of all German institutions listed by DEGRO) and absolute counts for individual platforms (e.g., Facebook, Instagram, LinkedIn). Institutional engagement or activity was defined as an institution’s active use of a given platform, indicated by at least one published post during the 6‑month observation period. This distinguishes engagement from mere presence, which refers solely to the existence of an account on a platform, regardless of its activity. The analysis employed pie charts to represent proportional differences and line graphs to track activity trends over time.
2. Posting frequency and rate: the number of posts made during the 6‑month period was examined, with a particular focus on differentiating between hospital-based facilities and medical care centers (MVZ). Frequency distributions were illustrated through histograms, allowing comparisons between institution types.
3. Content categories were represented using stacked bar charts, showing both the overall distribution and platform-specific trends. Posts were classified into six thematic areas:
  Project and clinical practice updates.
  Scientific studies and research findings.
  Events and announcements.
  Job announcements and recruitment.
  Health awareness and educational content.
  Team news.
4. Follower and post distributions were analyzed across the different social media platforms using scatter plots to depict the relationship between follower number and activity level for each institution. For manual posts count, we reviewed each post made within the past 6 months and categorized it accordingly. Each post was read and assigned to one of the predefined content categories. Regarding the follower count, we directly extracted these numbers from the account descriptions on the respective platforms, as these figures are typically provided by the platforms themselves.

The data were then analyzed using Microsoft Power BI Desktop, version 2.139.1678.0 (Microsoft Corporation, Redmond, WA, USA). The analysis involved entirely descriptive statistics to summarize and categorize the content into predefined themes, and no advanced statistical tests were applied.

## Results

### Institutional presence and engagement analysis

**Fig. 1:**
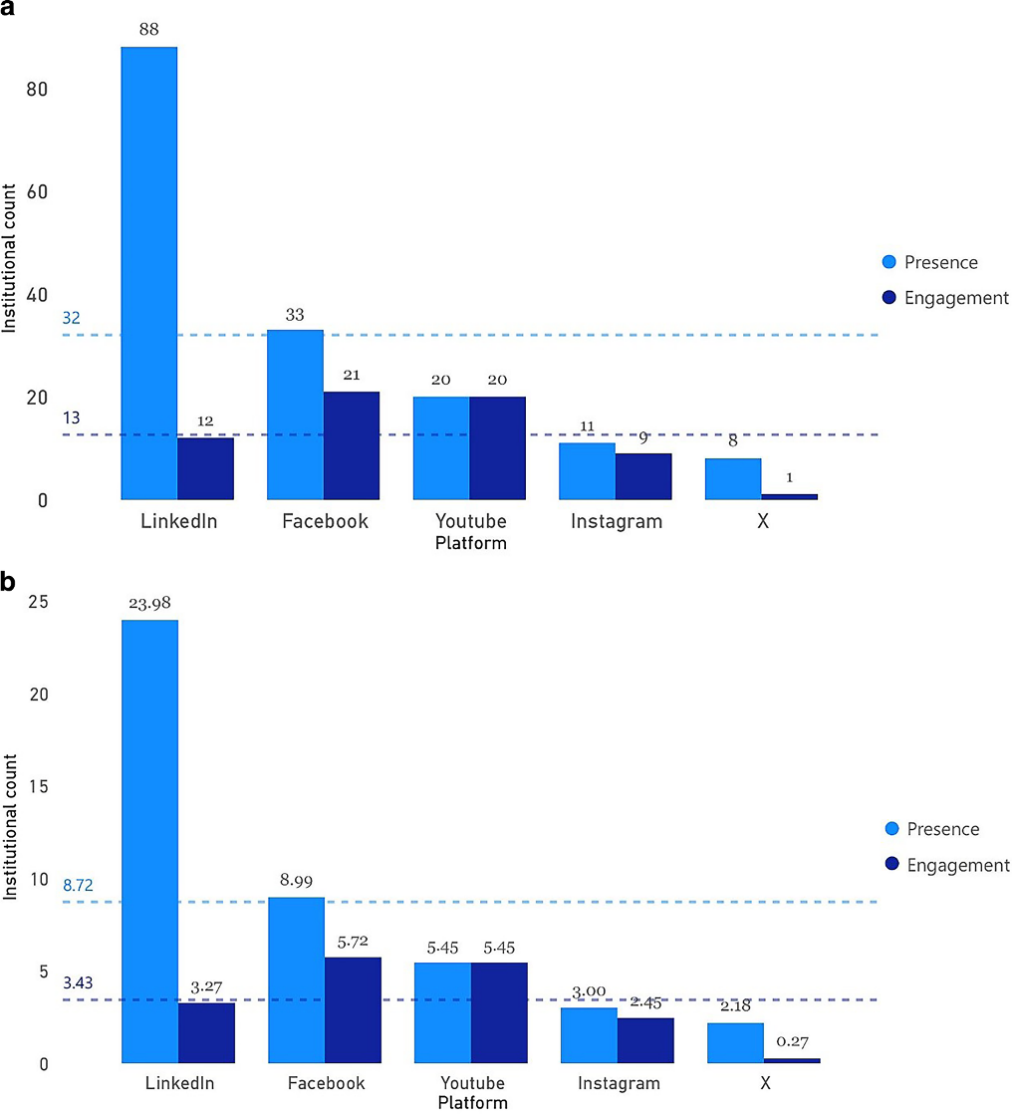
**a** Analysis of institutional presence and engagement across social media platforms in absolute values; **b** analysis of institutional presence and engagement across social media platforms in relative values

Figure 1 represents a comparative analysis of institutional presence and engagement on different social media platforms (LinkedIn, Facebook, YouTube, Instagram, and X) using absolute and relative values. Figure 1a illustrates the count of institutions active on each platform. LinkedIn dominates with the highest presence (88 institutions) and engagement (12 institutions). Facebook ranks second, with 33 institutions in presence and 21 in engagement. YouTube and Instagram show similar absolute metrics, with each platform having presence and engagement values ranging between 20 and 11 institutions. Figure 1b shifts focus to relative values, which consider the data in relation to the total number of institutions listed by DEGRO (367 institutions). LinkedIn leads with a high relative presence (24%) and engagement (3%). Facebook follows with relative values of 9% (presence) and 6% (engagement). YouTube and Instagram exhibit comparable relative values, with both platforms achieving approximately 6% for presence and engagement on YouTube and 3% for presence on Instagram. X has the lowest relative metrics, with a presence of 2% and engagement of 0.27%, indicating minimal relevance for institutional activities.

### Frequency of posts and content categories

**Fig. 2:**
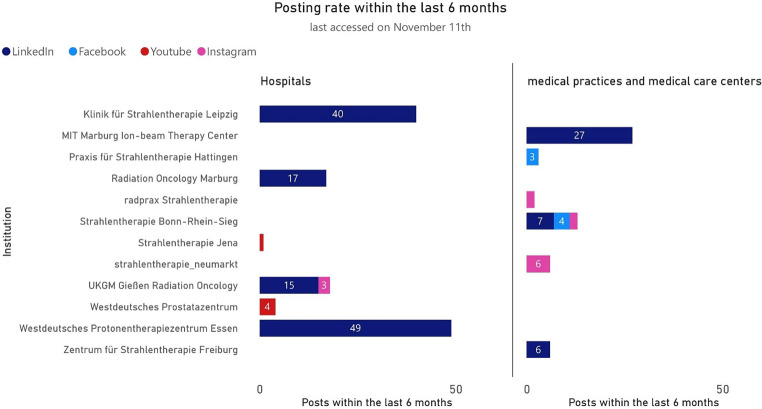
Frequency of posts of hospitals and medical practices/medical care centers on four social media platforms

Figure 2 illustrates the frequency of posts of hospitals and medical practices/medical care centers on four social media platforms (LinkedIn, Facebook, YouTube, and Instagram) over the previous 6 months during the observation period. Platform X is excluded from this analysis, as no posts were published on X during the observed period. In the hospitals category, the Westdeutsches Protonentherapiezentrum Essen (WPE), as part of the Universitätsmedizin Essen, is the most active institution, with 49 LinkedIn posts. The Klinik für Strahlentherapie Leipzig follows with 40 LinkedIn posts. Institutions like Radiation Oncology Marburg (17 LinkedIn posts) and UKGM Gießen Radiation Oncology (15 LinkedIn posts and 3 Instagram posts) also display moderate activity. A few institutions, such as Strahlentherapie Jena, utilize platforms like YouTube selectively, posting once during the analyzed period. For medical practices and medical care centers, the Zentrum für Strahlentherapie Freiburg leads with 27 LinkedIn posts, followed by Strahlentherapie Bonn-Rhein-Sieg, which posts across LinkedIn (7 posts) and Facebook (4 posts).

**Fig. 3:**
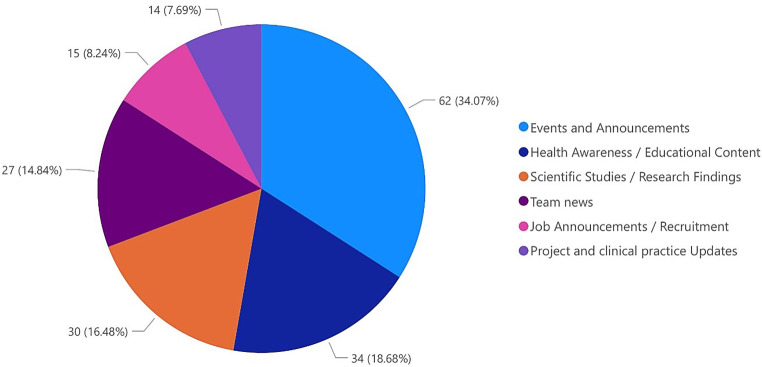
Distribution of content categories in social media posts as a total overview

Figure 3 illustrates the distribution of content categories in social media posts by institutions. The data highlight trends in communication priorities across institutional social media strategies. The largest share of posts (62 posts, 34%) belongs to the category “events and Announcements,” suggesting that institutions predominantly focus on promoting upcoming events, achievements, or important updates. Following this, “health awareness and educational content” accounts for 34 posts (19%), emphasizing the significance of public health education and engagement. The third-largest category is “scientific studies and research findings,” which comprises 30 posts (16%). “Team news,” with 27 posts (15%), highlights a focus on internal updates such as staff achievements, team expansions, or personal milestones. Meanwhile, “job announcements and recruitment,” with 15 posts (8%), reflects a moderate emphasis on attracting talent and sharing career opportunities. Finally, “project and clinical practice updates,” with 14 posts (8%), represents the smallest category.

### Content of posts and number of followers across platforms

**Fig. 4:**
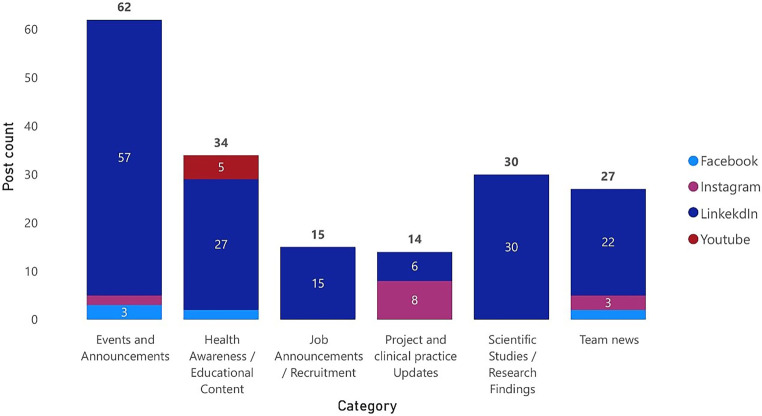
Social media content distribution across platforms and categories

Figure 4 provides an overview of how institutions distribute their social media posts across different content categories and platforms, including Facebook, Instagram, LinkedIn, and YouTube. The data highlight the dominant role of LinkedIn in nearly all content categories, while other platforms like Instagram, Facebook, and YouTube play secondary or niche roles.

In the category of “job announcements and recruitment,” all 15 posts are exclusively on LinkedIn, reflecting its status as the leading platform for professional networking and recruitment activities. “Project and clinical practice updates,” totaling 14 posts, show a more balanced distribution across platforms, with LinkedIn and Instagram each contributing 6 posts, and YouTube accounting for 2 posts. For “scientific studies and research findings,” all 30 posts were exclusively shared on LinkedIn, reinforcing its importance for reaching academic and professional audiences with research-driven content. Finally, “team news,” with 27 posts, also relies heavily on LinkedIn (22 posts), with smaller contributions from Instagram (3 posts) and Facebook (2 posts). This category highlights the focus on communicating team updates and achievements through professional channels.

**Fig. 5:**
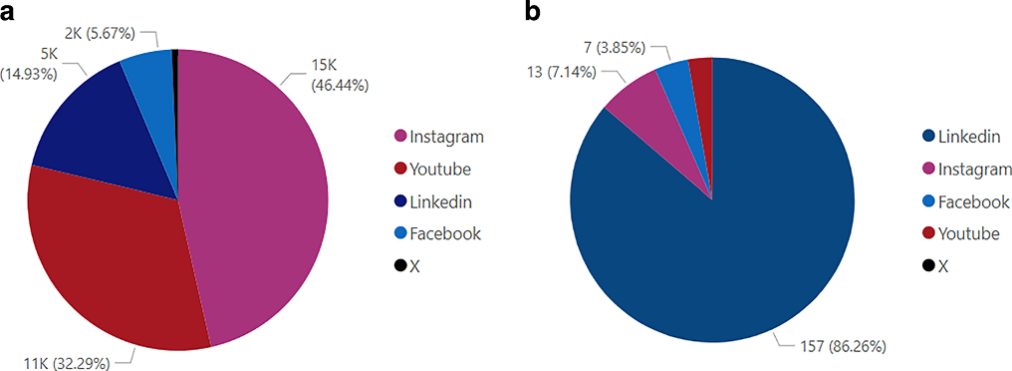
Follower (**a**) and post (**b**) distribution within the previous 6 months across social media platforms. Last accessed on November 11, 2024

In Fig. 5, an analysis of follower distribution and posting activity across various social media platforms is provided. In Fig. 5a, it can be seen that Instagram holds the largest share of followers, with 15,000 followers (46%). LinkedIn follows with a total of 11,000 followers (32%), indicating its significance as a professional networking platform. YouTube accounts for 5000 followers (15%), while Facebook has a smaller share, with 2000 followers (6%). X has a small share, with 218 followers (1%). Fig. 5b shows that LinkedIn is the most active platform, with 157 posts (86%), reflecting its central role in institutional communication. Instagram follows with 13 posts (7%), while Facebook and YouTube have minimal activity, with 7 (4%) and 5 posts (3%), respectively. No posts were recorded on X during this period, showing it to be an inactive platform for institutional use.

## Discussion

To our knowledge, this is the first report with a detailed analysis of SoMe content from German radiotherapy facilities. The results provide an overview of the social media presence and engagement of German radiotherapy institutions, focusing on platform activity, follower distribution, posting frequency, and content prioritization. The analysis examines variations between larger hospitals and smaller practices, highlighting key trends in platform preference and communication strategies.

Overall, around a quarter of all listed institutions have a SoMe account according to our analysis. In terms of the postings measured, larger facilities, especially university hospitals, are more active than smaller radiotherapy facilities. However, even among the larger facilities, we observed significant differences in the frequency of posts across the various institutions. Some institutions post nearly twice a day, while others share content only once a month at most. These variations can be attributed to several factors, and one important aspect is the level of investment in dedicated social media management.

Certain institutions have recognized the importance of a strategic, consistent social media presence and have hired personnel specifically for this purpose. These social media managers are tasked with generating content ideas, implementing them, and regularly posting on the institution’s social media accounts. This approach is part of a comprehensive social media strategy that ensures a professional and coherent online presence, with content often planned and scheduled in advance.

In contrast, other institutions take a more flexible approach, where the responsibility for social media activity is shared among various staff members. Here, social media posts are often created during working hours by individuals who may not have a specific mandate for social media management. In some cases, departmental heads or senior staff post on an ad hoc basis, whenever they have time or/and something of value to post and share. This less structured approach can result in sporadic posting activity, with less consistency in terms of frequency and content.

These differences in strategy are significant because they impact the visibility and overall activity of institutional social media accounts. While institutions with dedicated social media teams may have a more active and structured presence, those relying on individuals to handle social media may appear less engaged, even though the staff members themselves may be highly active online through personal accounts or other means.

Our key findings from the analysis presented are as follows:

- Of all analyzed platforms, LinkedIn shows the highest institutional presence and engagement, while X ranks lowest in both absolute and relative metrics.
- Concerning the frequency of posts, the Westdeutsches Protonentherapiezentrum Essen (WPE) is the most active institution, followed by the Klinik für Strahlentherapie Leipzig.
- Content categories: the majority of posts focus on events & announcements, followed by health awareness (19%), scientific studies (16%), and team news (15%), while job postings (8%) and clinical updates (8%) are less prominent.
- Content distribution across platforms: LinkedIn dominates nearly all content categories, including job announcements, scientific studies, and team news. Other platforms like Instagram, Facebook, and YouTube have niche roles, with LinkedIn taking the lead for professional and research-driven content, at least during the observation period.
- Follower and posting activity: Instagram has the largest follower share (46%), but LinkedIn remains the most active platform, accounting for 86% of posts.

There is at least one notable study that has taken a similar approach, but it did not exclusively examine the activity of institutional accounts. The study by Prabhu et al. from 2021 showed that there had been a recent significant growth of SoMe activity [10]. They analyzed all public tweets with the hashtag #radonc in the period 2014–2019. As a result, they found an increase from 23 to 119 countries from which tweets were made. The most frequent posts came from the USA, the UK, and Spain. They recorded an annual growth rate in Twitter/X users of 70.5%, with doctors responsible for the most tweets, at 46.9%. Looking at the annual number of tweets by origin, doctors and scientists are in the top two positions, but healthcare providers such as hospitals and medical practices (health care providers) also showed significant annual growth rates of 92.4% on average.

There have already been some analyses of the use of SoMe by health care professionals, which have revealed insights into user behavior. In a survey on the topic of SoMe use by radiation oncologists (RO) and physicists within the scope of the Catalan-Occitan Oncology Group (GOCO) for example, it was shown that it is quite common for doctors and physicians to change their clinical practice following information in SoMe (28%) [1]. The platforms Instagram (*n* = 116) and Facebook (*n* = 107) were primarily used, Twitter less frequently (*n* = 77).

In a further survey among a group of the European Heart Rhythm Association (EHRA), it was reported that most users are passive (38.3%), and only 19% actively post or share content [2]. On average, users spent around 5 h per week on SoMe. When asked about the reasons for using SoMe, interest in new publications (66%) and networking (48.5%) were cited.

While the German institutional accounts predominantly post information on events and announcements in our analysis, the topics “health awareness” and “scientific studies” are lagging behind in second and third place, respectively. In the work by Prabhu et al. discussed above, the top topic in the 2014–2016 period was “promotion of #radonc community,” which then slipped to seventh place in the following years.

In the more recent time period from 2017 to 2019, the topics “content around radiation” and “general discussions and content” were ranked first and second. However, it should be noted that the authors analyzed all tweets worldwide, primarily from individuals, whereby it is obvious that institutional accounts are more likely to post job advertisements, team news, or further promotion-related topics.

This study focused exclusively on institutional social media accounts to evaluate how radiotherapy institutions use these platforms for professional communication. By analyzing official profiles—typically used to share scientific achievements, institutional news, and events—we aimed to gain insight into the strategies institutions employ to maintain a professional digital presence. While educational content is sometimes initiated by individual professionals, personal accounts often cover broader topics beyond the scope of institutional communication.

However, the study does have several limitations. First, the 6‑month observation period provides only a snapshot, potentially missing long-term trends or seasonal variations. Second, our approach was primarily descriptive, limiting the ability to draw causal conclusions. Third, some institutions may have been unintentionally excluded due to limitations in our search criteria. Lastly, the study focused on quantitative metrics (e.g., number of posts and followers), without analyzing qualitative engagement, such as comments or interactions, which could offer deeper insight into audience engagement and impact.

## Conclusion

The analysis reveals that 24% of German radiotherapy institutions maintain active social media profiles, with larger hospitals being more active than smaller institutions. To date, LinkedIn represents the most frequently used platform, serving as the dominant channel for events, scientific publications, health awareness, recruitment, and team news. Facebook and YouTube are used as niche platforms for specific content categories, whereas X shows minimal relevance in institutional social media strategies. This distribution reflects a strategic allocation of resources, balancing professional engagement with broader public outreach.

## Supplementary Information

Suppl. Table 1

Suppl. Fig. 1 Geographical distribution of radiotherapy institutions across German federal states

Suppl. Fig. 2 Proportional distribution of radiotherapy institutions across German federal states
