# Supplementary material for: From radiation beams to digital streams: social media engagement of German radiotherapy facilities
Source: Strahlenther Onkol. 2025 Jul 17;202(6):627–34. doi: 10.1007/s00066-025-02439-3 (PMC13216080; doi:10.1007/s00066-025-02439-3)
Supplement: Supplementary file 1 — Suppl. Table 1 [file 66_2025_2439_MOESM1_ESM.pdf]

LinkedIn	X	Instagram	Facebook	Youtube
88	8	11	33	20
Klinik und Poliklinik für Strahlentherapie Leipzig	Strahlentherapie	radprax_strahlentherapie	GEmeinschaftspraxis für Strahlentherapie Hannover	Strahlentherapie
Strahlentherapie Bonn-Rhein-Sieg	Strahlentherapie	strahlentherapiebonnrheinsieg	Strahlentherapie Süd	Die Strahlendocs
Strahlentherapie Nord	Strahlentherapie	strahlentherapie_helle_mitte	Strahlentherapie Zentren Dr. Med. Adrian Staab	Strahlentherapie Dr. Adrian Staab
Zentrum für Strahlentherapie Freiburg	Die Strahlentherapie	strahlentherapiesud	STM Praxis für Strahlentherapie Mannheim	Strahlentherapie Jena
Strahlentherapie Bergedorf/Harburg	Strahlentherapie Leipzig	gsr_strahlentherapie	Strahlentherapie Bonn-Rhein-Sieg	Strahlentherapie Moabit
Strahlentherapie-Minden-Schaumburg	inik für Strahlentherapie/Radioonkologie Ul	dtzstrahlentherapie	Gemeinschaftspraxis für Strahlentherapie	Strahlentherapie Harburg
MVZ Strahlentherapie	Strahlentherapie Bergedorf	strahlentherapie_neumarkt	Strahlentherapie Kaufbeuren	Strahlentherapie Neustadt
Strahlentherapie Frechen	Strahlentherapie B73	strahlentherapie_harburg	Praxis für Strahlentherapie Hattingen	Strahlentherapie Nord
Strahlentherapie Harburg		strahlentherapie_gmuend	Strahlentherapie Wesel	Strahlentherapie Hattingen Dr. med. Daniel Metzler
Strahlentherapie RheinMainNahe		diestrahlendocs	Strahlentherapie	RadioChirurgicum GmbH CyberKnife®Südwest
Strahlentherapie Zentrum Bochum		g.ukgm_radiation_oncology	X care Strahlentherapie	Tumor Target Therapy
Praxis für Strahlentherapie Bergisch Gladbach			DTZ Strahlentherapie	Strahlenzentrum Hamburg 2.0
Strahlentherapie Schwäbisch Gmünd			Strahlentherapie Frank Muckelbauer in Neumarkt	Westdeutsches Prostatazentrum
diacura-coburg-abtl-strahlentherapie			Strahlentherapie-Sued	Strahlenzentrum Hamburg MVZ
Strahlentherapie Zentrum Harburg			curavid - Praxis für Strahlentherapie und Radiologie	Dr. Dalia Moustafa-Hubmer
Gemeinschaftspraxis für Strahlentherapie			Zentrum für Strahlentherapie Freiburg	Henrik Schachner
Curavid Strahlentherapie und Radiologie			MVZ Strahlentherapie Köln	
MVZ Strahlentherapie Halle			Strahlentherapie Harburg-Bergedorf	CDT WEST
Strahlentherapie in Moabit			MVZ Strahlentherapie Rhein Main Nahe GmbH	Xcare Gruppe
Strahlentherapie Nordhorn			Strahlentherapie und Radioonkologie Innsbruck	
Strahlentherapie 360 Aachen			Strahlentherapie-Bocholt	Klinik für Urologie und Uroonkologie Magdeburg
Kliniken Maria Hilf Klinik für Strahlentherapie			Strahlentherapie LMU	RadioOnkologie im Vosspalais
MVZ Strahlentherapie Frankfurt			Strahlentherapie (Radiotherapie) für Hund und Katze in der Tiermedizin	
Strahlentherapie Herr Dr				
Strahlentherapie Bonn Rhein			Gemeinschaftspraxis für Strahlentherapie	
Strahlentherapie Hauptkooperationspartner			Strahlentherapie Osnabrück	
Klinik und Poliklinik für Strahlentherapie			UKGM Marburg Strahlentherapie	
Strahlentherapie Duisburg Moers				
Strahlentherapie Pforzheim			Strahlentherapie Rhein/Pfalz	
Strahlentherapie Meppen - Dres med. Davar Hatami			Praxis für Radiologie und Strahlentherapie in Lindau	
Strahlentherapie Hattingen Dr			Zentrum für Strahlentherapie	
Strahlentherapie Böblingen				
Strahlentherapie Distler			Meine Strahlentherapie	
strahlentherapie-mtk			Gemeinschaftspraxis für Strahlentherapie Essen	
ntherapie-dres.med.-ostkamp-b%C3%B6lling-und-partner-radiologie			Strahlentherapie Minden-Schaumburg	
Klinikum Aschaffenburg Abt. für Strahlentherapie			Klinik für Strahlentherapie an der Uniklinik Köln	
Klinikum Traunstein Abteilung für Strahlentherapie				
Praxis für Strahlentherapie Helle Mitte				
entherapie Wesel Med. Versorgungszentrum am Ev. Krankenhaus GmbH				
aftspraxis Strahlentherapie Bonn-Rhein-Sieg Standort St. Marien-Hospital				
Praxis für Strahlentherapie Neustadt				
Dr.med. Steffen Hennies Facharzt für Strahlentherapie				
diologie Nuklearmedizin Strahlentherapie Dr. Ginter, Ilgemann,				
Praxis für Radioonkologie und Strahlentherapie				
linikum Hanau Klinik für Radioonkologie und Strahlentherapie				
Medizinische Klinik Strahlentherapie				
Christoph Güßbacher. Strahlentherapie				
MVZ Med. Versorgungszentrum Strahlentherapie Nürnberg				
sches Netzwerk Rheinland, Strahlentherapie am Marienhospital Aachen				

axis für Strahlentherapie Trier-Ehrang Xcare Dres. Eva-Maria Freitag Guido Syré u.w.
St. Josef-Hospital Strahlentherapie
Klinikum Dortmund Klinik f. Strahlentherapie
Dilek Kümbet Fachärztin f. Strahlentherapie
Dr. med. Jörg Schäfer Strahlentherapie
Sven Stolzenberg Facharzt für Strahlentherapie
Friederikenstift Ev. Krankenhaus Abt. für Strahlentherapie
Klinikum St. Marien Klinik für Strahlentherapie
Städt. Klinikum Görlitz - Klinik für Strahlentherapie
SRH Zentralklinikum Suhl Klinik für Strahlentherapie
Dr.med. Michael Klug Facharzt für Strahlentherapie
Dr.med. Heike Stattaus Fachärztin f. Strahlentherapie
Dr.med. Johann Meier Facharzt für Strahlentherapie
Dr.med. Jörg Distler Facharzt für Strahlentherapie
Dr.med. Alfred Haidenberger Facharzt für Strahlentherapie
Dr.med. Hanno Koppe Facharzt für Strahlentherapie
Dr.med. Ralf Kurek Facharzt für Strahlentherapie
Dr.med. Dorothea Riesenbeck Fachärztin f. Strahlentherapie
Dr.med. Stephan Hennings Facharzt für Strahlentherapie
Dr.med. Gregor Spira Facharzt für Strahlentherapie
Dr.med. Hans-Werner Anton Facharzt für Strahlentherapie
PD Dr.med. Ralf Wilkowski Facharzt für Strahlentherapie
med. Klaus Ostkamp-Morgenthaler Facharzt für Strahlentherapie
Dr. med. Ralf Rohn Praxis für Strahlentherapie
Prof.Dr.med. Ulrich M. Carl Facharzt für Strahlentherapie
Dr.med. Irene Speiser-Held Fachärztin f. Strahlentherapie
Dipl.-Med. Julich Dr.med. Schirm Praxis für Strahlentherapie
reas Koziorowski Med. Versorgungszentrum Facharzt für Strahlentherapie
ichael Glag Dr. Eckehard H. u. Dr. med. Heike Stattaus Strahlentherapie
AD überörtl.Gemeinschaftspraxis für Radiologie, Nuklearmedizin und Strahlentherapie
, A., Dr., Schüler, F., Dipl.-Med. u. Dr. M. Steingräber Strahlentherapie
Radiation Oncology Marburg
UKGM Gießen Radiation Oncology
National Center for Tumor Diseases (NCT) Heidelberg
Westdeutsches Protonentherapiezentrum Essen (WPE)
ManagedPhysics GmbH
IMI - Individualised Multimodal Immunotherapy for cancer
mitparticletherapycenter
uct-university-cancer-center-frankfurt
