# Supplementary material for: From radiation beams to digital streams: social media engagement of German radiotherapy facilities
Source: Strahlenther Onkol. 2025 Jul 17;202(6):627–34. doi: 10.1007/s00066-025-02439-3 (PMC13216080; doi:10.1007/s00066-025-02439-3)
Supplement: Supplementary file 2 [file 66_2025_2439_MOESM2_ESM.odt]

A map of germany with orange dots

Description automatically generated

Suppl. 1 Geographical distribution of radiotherapy institutions across German federal states
