# Supplementary material for: From radiation beams to digital streams: social media engagement of German radiotherapy facilities
Source: Strahlenther Onkol. 2025 Jul 17;202(6):627–34. doi: 10.1007/s00066-025-02439-3 (PMC13216080; doi:10.1007/s00066-025-02439-3)
Supplement: Supplementary file 3 [file 66_2025_2439_MOESM3_ESM.odt]

A screenshot of a graph

Description automatically generated

Suppl. 2 Proportional distribution of radiotherapy institutions across German federal states
